# Intracellular levels of hepatitis B virus DNA and mutational patterns of the polymerase gene of hepatitis B virus in peripheral blood mononuclear cells of patients with lamivudine or telbivudine resistance

**DOI:** 10.3892/etm.2014.2156

**Published:** 2014-12-24

**Authors:** XIAOGUO ZHANG, LU LIU, WANSU XU, YUN WANG, SHUCHUN GAO, SHIJUN CHEN, YIZHEN DU

**Affiliations:** 1 Division of Liver Disease, Jinan Infectious Disease Hospital, Shandong University, Shandong 250021, P.R. China; 2 Shandong University School of Medicine, Jinan, Shandong 250021, P.R. China

**Keywords:** hepatitis B virus, peripheral blood mononuclear cells, hepatitis B virus DNA, lamivudine, telbivudine, resistance, mutation pattern

## Abstract

Studies are limited regarding the association between the quantity of hepatitis virus B (HBV) DNA loads in serum and peripheral blood mononuclear cells (PBMCs) in patients with drug resistance and few studies focus on the mutational pattern of the polymerase gene of HBV in PBMCs of patients with drug resistance. The aim of the present study was to investigate the association between the quantity of HBV DNA loads in serum and PBMCs in patients with lamivudine (LAM) or telbivudine (LdT) resistance and to explore the mutational pattern of the polymerase gene of HBV in PBMCs of these patients. A total of 51 patients underwent antiviral therapy with LAM or LdT for at least half a year. The present study applied quantitative polymerase chain reaction for the quantification of total HBV DNA, and direct sequencing was used to detect the mutation. In total, 31 patients (60.78%) were detected to have drug resistance. The mean serum HBV DNA levels were significantly higher than the HBV DNA levels of PBMCs, whether in patients with LAM or LdT resistance. The PBMCs HBV DNA loads were correlated with the serum HBV DNA loads in the two groups. Three and two mutational patterns were found in the serum of patients with LAM and LdT resistant, respectively. In total, 85.71% of patients with LAM resistance and 75.00% of patients with LdT resistance presented the same mutational pattern in paired PBMCs and serum. The HBV DNA levels in the PBMCs of patients with LAM or LdT resistance were significantly lower than those in serum and there were positive correlations between them. The majority of the mutational patterns of the polymerase gene of HBV DNA in PBMCs were the same as those in the paired serum. These findings may help to increase knowledge regarding HBV drug resistance.

## Introduction

Infection of hepatitis B virus (HBV) remains a global health problem. Patients with chronic hepatitis B (CHB) can develop progressive liver disease, which can result in cirrhosis and hepatocellular carcinoma (HCC). These stages of the disease are associated with an increased risk of morbidity and mortality, and incur considerable healthcare costs (1). To reduce morbidity and mortality from chronic HBV infection, antiviral treatment is the only effective approach (2). Current antiviral agents for the treatment of CHB include interferon (IFN) and nucleoside analogues (NUCs). Although treatment with IFN may lead to a durable response, the unpleasant adverse effects and high cost limit its use (3). NUCs, including lamivudine (LAM) and telbivudine (LdT), then become the most common drugs used for antiviral therapy (4). Currently, LAM, adefovir, entecavir (ETV), tenofovir and LdT have been licensed for the treatment of CHB (5,6). NUCs target the HBV reverse transcriptase (RT), thus inhibiting viral replication and leading to virological, biochemical and histological improvement in the majority of patients. Current therapy of CHB does not eradicate HBV and has limited long-term efficacy, which results in the requirement for long-term therapy. Emergence of drug-resistant HBV mutants is an adverse consequence of long-term therapy (7). The high mutational rate of HBV RT enzyme, due to its lack of proof-reading activity, is the main cause of HBV mutation occurrence and drug resistance (8). Mutations selected by treatment with NUCs can be separated into two groups: Those that cause resistance, sometimes leading to decreased viral fitness; and compensatory mutations, which partially or fully restore viral fitness (9). LAM is the first safe oral NUC approved for the treatment of HBV by the Food and Drug Administration (10). LAM and LdT belong to L-nucleosides. The main mutation associated with L-nucleoside resistance is rtM204I/V, a mutation that occurs within the YMDD motif of the RT region of the polymerase (11).

Previous studies have shown that HBV infection exists in peripheral mononuclear cells (PBMCs) (12–14), and HBV viral loads in PBMCs correlated with serum HBV DNA (15). There is limited knowledge regarding the association between the quantity of HBV DNA loads in serum and PBMCs in patients with drug resistance. Although a number of studies have investigated the characteristics of resistance mutations of HBV in serum (4,6,16), few studies focus on the mutational pattern of the polymerase gene of HBV in PBMCs. In the present study, quantitative polymerase chain reaction (PCR) was applied for the quantification of total HBV DNA in serum and PBMCs in patients with LAM or LdT resistance, and direct sequencing was used to detect the mutation of the polymerase gene of HBV. Clarification of the association between serum and PBMC viral loads was attempted, and whether there were different mutational patterns of the polymerase gene of HBV in serum and PBMCs of patients with LAM or LdT resistance was explored.

## Materials and methods

### Patients and samples

The study was approved by the Ethics Committee of the Jinan Infectious Disease Hospital, Shandong University (Jinan, China), and written informed consent was obtained from all the participants. Between January 2012 and January 2013, 51 patients with CHB were recruited from the Hepatitis Clinic of the Jinan Infectious Disease Hospital of Shandong University. All the patients were on antiviral therapy with LAM (100 mg/day; GlaxoSmithKline Biological Co., Ltd., Shanghai, China) or LdT (600 mg/day; Novartis Pharma Co., Ltd., Beijing, China) for at least half a year and experienced a virological breakthrough (HBV DNA level increased by 1 log_10_ copies/ml or more than the treatment nadir). The patients were divided into two groups: LAM (n=32) and LdT (n=19). All the patients were positive for hepatitis B surface antigen (HBsAg), had HBV viral loads ≥ 3 log_10_ copies/ml, and did not have other infectious diseases, including human immunodeficiency virus, hepatitis C or hepatitis D. The venous blood samples were collected from the antecubital fossa of each participant. Serum was separated, divided into aliquots and maintained frozen at −80°C until testing. PBMCs were isolated using Lymphocyte Separation medium (Tianjin Haoyang Biological Technology Co., Ltd., Tianjin, China) and stored at −80°C.

### Testing for HBV serological markers and biochemical parameters

Serological markers for HBV infection were measured using micro-particle enzyme immunoassay with reagents from Abbott Laboratories (Abbott Park, IL, USA), and serum biochemical parameters were also detected (Abbott Laboratories).

### Quantitative determination of HBV DNA in serum and PBMCs

HBV DNA in serum was detected by the fluorescence quantitative PCR assay (HBV PCR Fluoroscence Diagnostic kit; Shenzhen PG Biotechnology Co., Ltd., Shenzhen, China). The lower limit of detection was 500 copies/ml, as according to the manufacturer’s instructions. PBMCs were washed three times in phosphate-buffered saline prior to DNA extraction, and the final cell wash was conserved as a control for contamination with HBV DNA derived from blood. Total HBV DNA in PBMCs was isolated according to the manufacturer’s instructions [TIANamp Genomic DNA kit; Tiangen Biotech (Beijing) Co., Ltd., Beijng, China]. HBV DNA in PBMCs was also detected by fluorescence quantitative PCR assay.

### Detection of HBV genotypes and mutations

HBV genotypes were detected by using the Genotypes Detection kit (Yuan Qi Bio-Medicine Co., Ltd., Shanghai, China) according to the manufacturer’s instructions. The mutations of the RT region of the polymerase gene of HBV were detected by Shanghai Shenyou Biotechnology Co., Ltd. (Shanghai, China). Briefly, a pair of primers was designed (forward, 5′-ATCCCATCATCTTGGGCTTT-3′; and reverse, 5′-CAA GGTACCCCAACTTCCAAT-3′) for amplification of the HBV polymerase region. PCR conditions were 95°C for 15 min, followed by 45 cycles consisting of 95°C for 45 sec, 56°C for 45 sec and 72°C for 45 sec. The PCR products were approximately 300 base pairs. All the PCR products were purified and directly sequenced. For cycle sequencing, the following thermal protocol was used: 35 cycles consisting of 95°C for 20 sec, 50°C for 25 sec and finally 60°C for 2 min. Data were accumulated by direct sequencing and were analyzed either manually or using the Chromas program (Chromas Lite version 2.1 (2012), Technelysium Pty Ltd, South Brisbane, Queensland, Australia).

### Statistical analysis

The results are expressed as percentages and the mean ± standard deviation. Differences between categorical variables were analyzed using the Fisher’s exact or χ^2^ tests. For continuous variables, the Student’s t-test was used when the data showed a normal distribution, or the Mann-Whitney U test was used when the data was not normally distributed. Pearson correlation was also used for normally distributed variables, and Spearman correlation for non-normally distributed variables. Values of P<0.05 (two-tailed) were considered to indicate a statistically significant difference. All the statistical processes were carried out by statistical software SPSS 13.0 for Windows (SPSS, Inc., Chicago, IL, USA).

## Results

### Characteristics of demographic, clinical and laboratory data

All the participants were ethnically Chinese and HBV genotype C positive. The aim was to investigate the mutational pattern of the polymerase gene of HBV in serum and PBMCs of patients with LAM or LdT resistance, therefore 20 patients (39.22%, including 11 patients in the LAM group and nine patients in the LdT group) were excluded when it was found that there were no drug resistance mutations in the serum and PBMCs of these patients. The characteristics of the remaining 31 patients with regard to demographic, clinical and laboratory data are shown in Table I. No significant differences were identified between the two groups.

### Comparison of HBV DNA levels of serum and PBMCs

**Figure 1:**
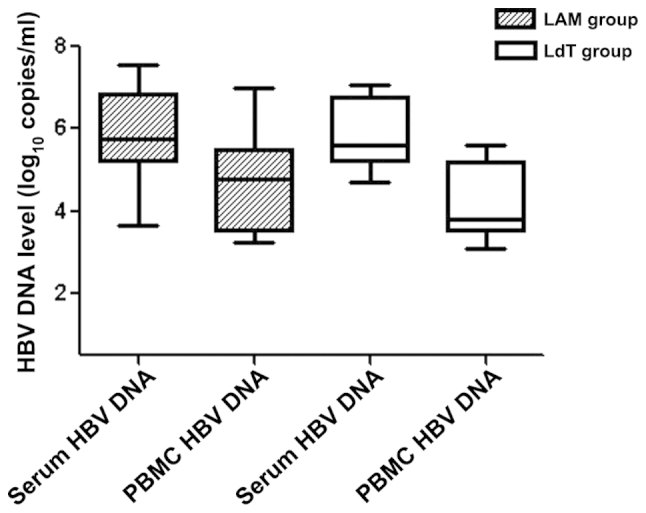
Serum and PBMC HBV DNA in the different groups. HBV, hepatitis B virus; PBMC, peripheral blood mononuclear cell; LAM, lamivudine; LdT, telbivudine.

HBV DNA loads of serum and PBMCs in the different groups are shown in Fig. 1 and Table II. No significant differences were observed between the two groups when comparing the HBV DNA loads in the serum and PBMCs, respectively (serum, t=0.15, P>0.05; PBMC, t=0.99, P>0.05). While analyzing the HBV DNA loads of serum and PBMCs, it was found that the HBV DNA level of serum was significantly higher than that of PBMCs in the two groups (LAM, t=5.69, P<0.05; LdT, t=9.87, P<0.05).

### Correlations between serum and PBMCs HBV DNA loads

**Figure 2:**
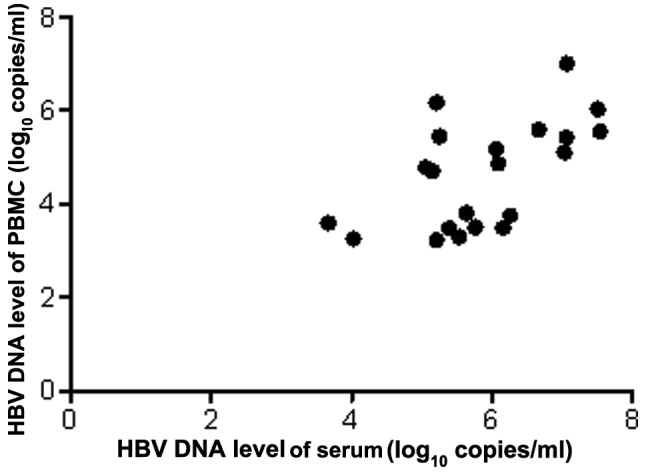
Correlations between serum and PBMC HBV DNA in the lamivudine group. PBMC, peripheral blood mononuclear cell; HBV, hepatitis B virus.

**Figure 3:**
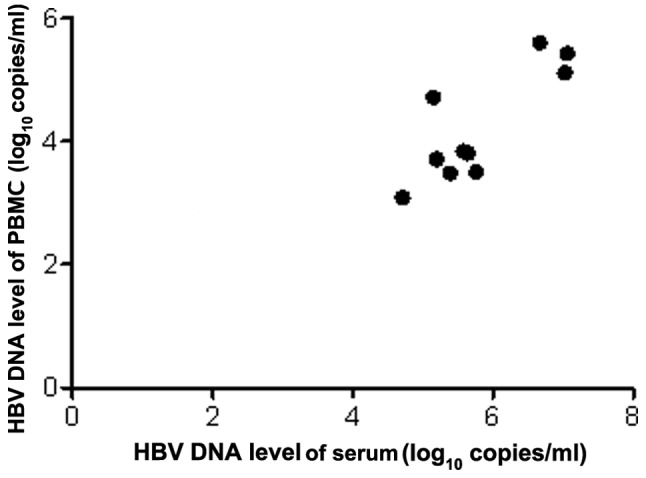
Correlations between serum and PBMC HBV DNA in the telbivudine group. PBMC, peripheral blood mononuclear cell; HBV, hepatitis B virus.

The HBV DNA loads of PBMCs were correlated significantly to that of serum, and there were positive correlations in the two groups (LAM, r=0.584, P<0.01; LdT, r=0.829, P<0.01). Scatterplots are shown in Figs. 2 and 3.

### Mutational patterns of the polymerase gene of HBV DNA in serum and PBMCs

Different mutational patterns in the HBV DNA polymerase gene were distinguished, as are shown in Table III. All the LAM resistant strains carried the mutation site, rt204, whether in serum or PBMCs. There were three mutational patterns in the serum of LAM-resistant patients: Single-site mutation (rtM204I, 3/21, 14.29%), two sites mutation (rtL180M plus rtM204I, rtM204V or rtM204I/V, 11/21, 52.38%) and three sites mutation (rtL180M plus rtM204I, rtM204V or rtM204I/V plus another site, 7/21, 33.33%). There were also three mutational patterns in the PBMCs of LAM-resistant patients. Analysis of the mutation sites in matched PBMCs and serum from each LAM-resistant patient revealed that 85.71% (18/21) patients had the same mutational pattern in the PBMCs and serum. Two mutational patterns were found in the serum of LdT-resistant patients: Single-site mutation (rtM204I, 8/10, 80%) and two sites mutation (rtL180M plus rtM204I, 2/10, 20.00%). Patients 7 and 8 of the LdT group had wild-type in PBMCs, whereas patients 9 and 10 had mutational strains in PBMCs, which showed a different mutational pattern from that in serum. Comparing with mutation sites in matched PBMCs and serum, 75% (6/8) of patients presented the same mutational pattern in the LdT group.

## Discussion

HBV is not strictly hepatotropic and studies have established that tissues from the kidney, pancreas and bone marrow, as well as PBMCs, contain HBV DNA sequences (17–19). Although hepatocytes are recognized as the main target, HBV has significant lymphotropic properties. HBV infection of lymphoid cells is an important mechanism whereby the virus escapes immune recognition and lymphoid reservoirs, particularly those harboring drug-resistant HBV, and may be key to the development of antiviral resistance (20). LAM was approved for the treatment of CHB in China in 1999. However, LAM is no longer recommended as a first-line agent for naïve patients with CHB due to its high incidence of drug resistance (5). A number of patients chronically infected with HBV remain treated with LAM due to cost reasons and availability (21). Therefore, the study of intracellular levels of HBV DNA and mutational patterns of the polymerase gene of HBV in PBMCs of patients with LAM resistance are clinically relevant.

Genotypes B and C of HBV have been identified as the most common strains and account for ~95% of infections among Chinese patients (22). Compared with HBV genotype B infections, HBV genotype C infections have been associated with lower rates of spontaneous clearance of HBsAg in serum, higher levels of virus replication, more advanced liver disease (23) and a lower rate of response to α-interferon therapy (24). All the subjects in the present study are genotype C, and all the HBV DNA in the PBMCs of the patients with LAM or LdT resistance was successfully detected. The HBV DNA loads in PBMCs were significantly lower than those in serum, whether in the LAM or LdT groups, and there were positive correlations between them. The present study showed the association between the quantity of HBV DNA loads in PBMCs and serum in patients with drug resistance conformed with those in patients without antiviral therapy (15). The reasons for the correlation between the viral load in serum and PBMCs were not clear. Although the HBV DNA loads in PBMCs are relatively low, it is difficult to eliminate HBV from PBMCs. Ke *et al* (14) found that the negative rate of HBV DNA in serum was 90.48%, but only 59.52% in PBMCs after 48 weeks of LAM treatment. Coffin *et al* (20) found HBV DNA was detected in 43% (3/7) of plasma, 100% (9/9) of liver and 83% (5/6) of PBMC samples using sensitive PCR/nucleic acid hybridization assay despite undetectable plasma HBV DNA by clinical assays following antiviral therapy. The study also found that PBMCs carried a drug-resistant virus in patients whose plasma had wild strains only. HBV can even persist in the serum and PBMCs for years after clinical and serological recovery from acute viral hepatitis, and remains transcriptionally active in PBMCs (25–27). HBV in PBMCs interferes with the immune activity of cells subsequent to HBV integrating with PBMCs, and decreases the contents of immunoglobulin, C3, tumor necrosis factor and activity of natural killer cells, as well as the ratio of cluster of differentiation 4^+^ (CD4^+^)/CD8^+^ (28). Therefore, the function of cell-mediated and humoral immunities are reduced and the curative effects of anti-viral drugs are affected.

As has been described previously, LAM and LdT belong to the L-nucleosides, therefore the study was also conducted on patients with LdT resistance. The main mutation associated with L-nucleoside resistance is rtM204I/V, a mutation that occurs within the YMDD motif of the RT region of the polymerase (11). The rtL180M mutation is the most common compensatory mutation, contributing to increasing either replication efficiency and/or antiviral resistance (29). In the present study, 60.78% (31/51) of patients who experienced a virological breakthrough during anti-viral therapy with LAM or LdT appeared to exhibit drug resistant strains in their serum. All the mutations associated with LAM or LdT resistance had the mutation site rt204. The majority of patients associated with LAM resistance had a compensatory mutation at position rt180 (18/21, 85.71%). Patients 13 and 14 in the LAM group had three mutation sites that were associated with ETV resistance despite those two patients never previously being administered ETV, which offered evidence as to why certain patients with LAM resistance did not experience beneficial effects when they changed to ETV for anti-viral therapy. Regarding the mutation patterns of HBV in PBMCs, 85.71% (18/21) of patients with LAM resistance and 75% (6/8) of patients with LdT resistance carried the same mutational pattern as that in the matched serum. In the LAM group, patients 12 and 15 had different mutational patterns between the PBMCs and serum despite the same mutation site: rtM204V/I in the PBMCs and rtM204V in the serum, while patient 17 carried one more mutation site in the PBMCs than in the serum. In the LdT group, patients 7 and 8 carrying mutants in the serum were detected to have wild strains in the PBMCs. According to the present results, it may be inferred that HBV in PBMCs may be wild-type at first, and gradually mutated following administration of the antiviral drug. The discrepancy of the mutational patterns between PBMCs and serum may be partly explained by the differences in antiviral drug selection pressure in the two compartments. HBV drug-resistant variants are present in PBMCs, therefore assessing drug resistance in a single compartment may not be sufficient in predicting the long-term response to antiviral therapy. Awareness of resistance patterns in PBMCs may help in antiviral therapy and predicting clinical outcomes.

In conclusion, the HBV DNA levels in the PBMCs of patients with LAM or LdT resistance were significantly lower than those in serum and there were positive correlations between them. The majority of the mutational patterns of the polymerase gene of HBV DNA in PBMCs were the same as those in the paired serum. These finding may help to increase knowledge regarding HBV drug resistance.

## Figures and Tables

**Table I:** Characteristics of patients in the different groups.

	Group			
			
Variable	Lamivudine	Telbivudine	t	P-value
Patients, n	21	10	-	-
Male, n (%)	18 (85.71)	8 (80.00)	-	1.00^a^
Age^b^, years	45.76±12.78	44.90±7.69	0.20	0.85
HBsAg positive, n (%)	17 (81)	9 (90)	-	1.00^a^
Treatment duration, months	27.00±17.69	20.30±9.32	1.12	0.27
ALT^b^, U/l	173.04±216.13	117.80±211.12	0.67	0.51
AST^b^, U/l	116.61±122.51	84.90±128.85	0.66	0.51

a Fisher’s exact test was used.

b Data are presented as the mean ± standard deviation.

ALT, alanine aminotransferase; AST, aspartate aminotransferase; HBsAg, hepatitis B surface antigen.

**Table II:** HBV DNA levels of serum and PBMCs in the different groups.

	HBV DNA (log_10_ copies/ml)	
	
Group	Serum	PBMCs
Lamivudine	5.87±1.04	4.63±1.14
Telbivudine	5.82±0.82	4.23±0.90

Data are presented as the mean ± standard deviation. HBV, hepatitis B virus; PBMCs, peripheral blood mononuclear cells.

**Table III:** Mutational patterns of reverse transcriptase of the polymerase gene in all the patients.

	Mutation sites of reverse transcriptase of the polymerase gene	
	
Groups and patients	Serum	Peripheral blood mononuclear cells
Lamivudine		
1	rtM204I	rtM204I
2	rtM204I	rtM204I
3	rtM204I	rtM204I
4	rtL180M + rtM204I	rtL180M + rtM204I
5	rtL180M + rtM204I	rtL180M + rtM204I
6	rtL180M + rtM204I	rtL180M + rtM204I
7	rtL180M + rtM204I	rtL180M + rtM204I
8	rtL180M + rtM204I + rtN238S	rtL180M + rtM204I + rtN238S
9	rtL180M + rtM204V	rtL180M + rtM204V
10	rtL180M + rtM204V	rtL180M + rtM204V
11	rtL180M + rtM204V	rtL180M + rtM204V
12	rtL180M + rtM204V	rtL180M + rtM204V/I
13	rtL180M + rtM204V + rtV173L	rtL180M + rtM204V + rtV173L
14	rtL180M + rtM204V + rtM250L	rtL180M + rtM204V + rtM250L
15	rtL180M + rtM204V + rtV207L	rtL180M + rtM204V/I + rtV207L
16	rtL180M + rtM204V + rtN238S	rtL180M + rtM204V + rtN238S
17	rtL180M + rtM204V/I	rtL180M + rtM204V/I + rtT184S
18	rtL180M + rtM204V/I + rtP237H	rtL180M + rtM204V/I + rtP237H
19	rtL180M + rtM204V/I	rtL180M + rtM204V/I
20	rtL180M + rtM204V/I	rtL180M + rtM204V/I
21	rtL180M + rtM204V/I + rtV173L	rtL180M + rtM204V/I + rtV173L
Telbivudine		
1	rtM204I	rtM204I
2	rtM204I	rtM204I
3	rtM204I	rtM204I
4	rtM204I	rtM204I
5	rtL180M + rtM204I	rtL180M + rtM204I
6	rtL180M + rtM204I	rtL180M + rtM204I
7	rtM204I	wild-type
8	rtM204I	wild-type
9	rtM204I	rtM204I + rtM250R
10	rtM204I	rtL180M + rtM204I + rtT184S

## References

[1] Lok AS, McMahon BJ (2009). Chronic hepatitis B: update 2009. Hepatology.

[2] Lavanchy D (2004). Hepatitis B virus epidemiology, disease burden, treatment, and current and emerging prevention and control measures. J Viral Hepat.

[3] Colacino JM, Staschke KA (1998). The identification and development of antiviral agents for the treatment of chronic hepatitis B virus infection. Prog Drug Res.

[4] Li SY, Qin L, Zhang L (2011). Molecular epidemical characteristics of Lamivudine resistance mutations of HBV in southern China. Med Sci Monit.

[5] European Association For The Study Of The Liver (2009). EASL Clinical Practice Guidelines: management of chronic hepatitis B. J Hepatol.

[6] Sayan M, Akhan SC, Senturk O (2011). Frequency and mutation patterns of resistance in patients with chronic hepatitis B infection treated with nucleos(t)ide analogs in add-on and switch strategies. Hepat Mon.

[7] Nguyen MH, Keeffe EB (2009). Chronic hepatitis B: early viral suppression and long-term outcomes of therapy with oral nucleos(t)ides. J Viral Hepat.

[8] Deng L, Tang H (2011). Hepatitis B virus drug resistance to current nucleos(t)ide analogs: Mechanisms and mutation sites. Hepatol Res.

[9] Sheldon J, Rodès B, Zoulim F, Bartholomeusz A, Soriano V (2006). Mutations affecting the replication capacity of the hepatitis B virus. J Viral Hepat.

[10] Josefson D (1998). Oral treatment for hepatitis B gets approval in the United States. BMJ.

[11] Bartholomeusz A, Locarnini SA (2006). Antiviral drug resistance: clinical consequences and molecular aspects. Semin Liver Dis.

[12] Pontisso P, Poon MC, Tiollais P, Brechot C (1984). Detection of hepatitis B virus DNA in mononuclear blood cells. Br Med J (Clin Res Ed).

[13] Liu MC, Wang GQ, Piao WH (2004). Detection of hepatitis B virus covalently closed circular DNA in peripheral blood mononuclear cells from patients with chronic hepatitis B infection. Zhonghua Gan Zang Bing Za Zhi.

[14] Ke CZ, Chen Y, Gong ZJ (2006). Dynamic changes of HBV DNA in serum and peripheral blood mononuclear cells of chronic hepatitis patients after lamivudine treatment. World J Gastroenterol.

[15] Lu L, Zhang HY, Yueng YH (2009). Intracellular levels of hepatitis B virus DNA and pregenomic RNA in peripheral blood mononuclear cells of chronically infected patients. J Viral Hepat.

[16] Hamidi-Fard M, Makvandi M, Samarbaf-Zadeh A (2013). Mutation analysis of hepatitis B virus reverse transcriptase region among untreated chronically infected patients in Ahvaz city (South-West of Iran). Indian J Med Microbiol.

[17] Murakami Y, Minami M, Daimon Y, Okanoue T (2004). Hepatitis B virus DNA in liver, serum, and peripheral blood mononuclear cells after the clearance of serum hepatitis B virus surface antigen. J Med Virol.

[18] Mason A, Wick M, White H, Perrillo R (1993). Hepatitis B virus replication in diverse cell types during chronic hepatitis B virus infection. Hepatology.

[19] Pontisso P, Morsica G, Ruvoletto MG (1991). Hepatitis B virus binds to peripheral blood mononuclear cells via the pre S1 protein. J Hepatol.

[20] Coffin CS, Mulrooney-Cousins PM, Peters MG (2011). Molecular characterization of intrahepatic and extrahepatic hepatitis B virus (HBV) reservoirs in patients on suppressive antiviral therapy. J Viral Hepat.

[21] Neumann-Fraune M, Beggel B (2013). High frequency of complex mutational patterns in lamivudine resistant hepatitis B virus isolates. J Med Virol.

[22] Wang Z, Huang Y, Wen S, Zhou B, Hou J (2007). Hepatitis B virus genotypes and subgenotypes in China. Hepatol Res.

[23] Pujol FH, Navas MC, Hainaut P, Chemin I (2009). Worldwide genetic diversity of HBV genotypes and risk of hepatocellular carcinoma. Cancer Lett.

[24] Kao JH, Chen PJ, Lai MY, Chen DS (2000). Hepatitis B genotypes correlate with clinical outcomes in patients with chronic hepatitis B. Gastroenterology.

[25] Michalak TI, Pasquinelli C, Guilhot S, Chisari FV (1994). Hepatitis B virus persistence after recovery from acute viral hepatitis. J Clin Invest.

[26] Rehermann B, Ferrari C, Pasquinelli C, Chisari FV (1996). The hepatitis B virus persists for decades after patients’ recovery from acute viral hepatitis despite active maintenance of a cytotoxic T-lymphocyte response. Nat Med.

[27] Cabrerizo M, Bartolomé J, Caramelo C, Barril G, Carreno V (2000). Molecular analysis of hepatitis B virus DNA in serum and peripheral blood mononuclear cells from hepatitis B surface antigen-negative cases. Hepatology.

[28] Pallier C, Castéra L, Soulier A (2006). Dynamics of hepatitis B virus resistance to lamivudine. J Virol.

[29] Xiong X, Yang H, Westland CE, Zou R, Gibbs CS (2000). In vitro evaluation of hepatitis B virus polymerase mutations associated with famciclovir resistance. Hepatology.

